# Alteration of T cell cytokine production in PLPp-139-151-induced EAE in SJL mice by an immunostimulatory CpG Oligonucleotide

**DOI:** 10.1186/1742-2094-8-59

**Published:** 2011-05-30

**Authors:** Vsevolod Smolianov, Thomas Dehmel, Patrick Vollmar, Anne K Mausberg, Bernd C Kieseier, Bernhard Hemmer, Hans P Hartung, Harald H Hofstetter

**Affiliations:** 1 Department of Neurology, Heinrich Heine University, Düsseldorf, Germany; 2 Department of Neurology, Klinikum rechts der Isar, Technische Universität München, Munich, Germany

## Abstract

Experimental autoimmune encephalomyelitis (EAE) is - in certain aspects - regarded as an animal model of the human CNS autoimmune disease multiple sclerosis (MS). While in EAE CNS-autoantigen-specific immunity is induced in a defined way, the initial processes leading to CNS autoimmunity in humans are so far unknown. Despite essential restrictions, which exist regarding the interpretation of EAE data towards MS, EAE might be a useful model to study certain basic aspects of CNS autoimmunity. Studies in MS have demonstrated that established autoimmune pathology can be critically influenced by environmental factors, in particular viral and bacterial infections. To investigate this interaction, EAE as an instrument to study CNS autoimmunity under defined conditions appears to be a suitable experimental tool. For this reason, we here investigated the influence of the Toll-like-receptor (TLR) ligand CpG oligonucleotide (CpG) on already established CNS autoimmunity in murine proteolipid protein (PLP)-induced EAE in SJL mice. CpG were found to co-stimulate PLPp-specific IFN-γ production in the peripheral immune system and in the CNS. However, CpG induced Interleukin (IL)-17 production in the inflamed CNS both alone and in combination with additional PLPp stimulation. These findings might indicate a mechanism by which systemic infections and the microbial stimuli associated with them may influence already existing CNS autoimmune pathology.

## Background

Research on the role of T cells in CNS autoimmune disease both in human diseases as well as in their experimental animal models currently centers on the endogenous requirements which are necessary for T cell activation as well as on the exogenous factors which trigger it. Among the environmental elements, which can influence this process (both in a positive and negative way), infections are considered important [1;2]. These include both viral and bacterial infections as well as the pathogenic factors which are associated with them. The potential role of microbial pathogens in triggering autoimmune disease has been extensively studied both in humans as well as in experimental animal models. As an example for one of many infectious agents which have been discussed as the cause of MS, a positive association between Epstein-Barr virus (EBV) infection and increased risk of developing MS thereafter has been broadly discussed [3]. Such clinical observations have been supported by basic observations from cell culture models which try to explain how infectious agents can affect the CNS and facilitate key steps in MS pathogenesis, e.g. through creating a local proinflammatory milieu in the early stages of disease [4]. In the EAE mouse model, there is currently increasing evidence for a critical role of commensal gut microbiota in the initiation of CNS autoimmunity, as demonstrated by experiments in which reduction of the commensal microflora by antibiotic treatment inhibits the development of EAE [5]. However, despite the fact that disease progression or relapse is clinically to the same extent associated with bacterial or viral infection as the beginning of disease, in particular in the case of MS [6-9], the influence of these pathogenic conditions on already established autoimmune disease has received less attention. In EAE in the SJL mouse model lipopolysaccharide, a TLR 4 ligand, has been shown to be able to induce relapses via antigen presenting cell (APC)-dependent activation of autoantigen-specific T cells [10].

When studying the impact of infection on ongoing CNS autoimmunity, a differentiated setup has to be chosen. First, systemic effects of a microbial stimulus on the autoimmune T cell population might differ from its effects in the CNS [11;12]. Second, different T cell populations might be affected in a distinct way, which might also be different in the periphery and in the inflamed CNS. T cell populations that are currently implied in CNS autoimmune pathology are Th1 and Th17 cells, which are characterized by the production of IFN-γ and IL-17, respectively [13]. Both cytokines are important mediators of disease and tissue damage in CNS autoimmunity, albeit with different roles in the autoimmune process and different resulting pathology [14-16].

For this reason, it was the purpose of this study to investigate the influence of CpG as a paradigm of a microbial stimulus which is able to activate both APC [17] as well as T cells directly [18] on the PLPp-specific T cell cytokine production in EAE in SJL mice, in particular in regard of IFN-γ and IL-17.

## Methods

### Animals, antigens and treatments

Female SJL/J mice at age 6-8 wk were purchased from Charles River (Sulzfeld, Germany) and maintained at the local animal facilities. Mice were divided in two groups and immunized subcutaneously either with 100 μg PLP peptide amino acids 139-151 (PLPp, Biotrend, Cologne, Germany) and 100 μg CpG oligonucleotide 1760 with the sequence ATAATCGACGTTCAAGCAAG (CpG, Eurofins MWG Operon, Ebersberg, Germany) in incomplete Freund's adjuvant (IFA, Difco, Detroit, USA) or with 100 μg PLPp in complete Freund's adjuvant (CFA, Difco, Detroit, USA). Mice immunized with PLPp in CFA were additionally given two intraperitoneal applications of 400 ng pertussis toxin (PTX, Sigma, Deisenhofen, Germany) in 500 μl phosphate buffered saline (PBS) on day 0 and day 2 after immunization. PLPp/CFA was prepared by mixing PLPp in CFA yielding a final concentration of 1 mg/ml. PLPp/CpG/IFA was prepared by mixing PLPp and CpG in IFA yielding a final concentration of 1 mg/ml for both agents. Mice were assessed daily for the development of paralytic symptoms, and the severity of disease was recorded according to a standard scale: grade 1, floppy tail; grade 2: hind leg weakness; grade 3: full hind leg paralysis; grade 4: quadriplegia; and grade 5: death. Access to food and drinking water was ensured for all mice, including those with paralysis. All animal experiments were approved by the local authorities for animal experimentation.

### Cell preparation from the organs tested

After sacrificing the animals with isoflurane euthanasia, spleens and spinal cords were prepared as described previously [19]. Subsequently, single cell suspensions were made. The cells were counted by trypan blue exclusion and plated at the cell numbers indicated in HL-1 medium (Lonza, Cologne, Germany).

### Adoptive transfer experiments

For adoptive transfer experiments, spleen cells were prepared from female SJL mice injected with PLPp/CFA and PTX as described above at day 7 after immunization. The resulting cells were incubated for three days in HL-1 medium at 37°C, 5% CO_2 _with PLPp (concentration 20 μg/ml) alone or with PLPp plus CpG (concentration 10 μg/ml). At the end of the incubation period cells were washed carefully several times and 40 × 10^6 ^cells were injected intraperitoneally per recipient mouse. Mice were assessed daily for the development of paralytic symptoms, and the severity of disease was recorded as described above.

### Stimulation regimens, cytokine measurements by ELISPOT and computer-assisted ELISPOT image analysis

ELISPOT assays were performed as described previously [20]. Briefly, MultiScreen _HTS_-IP plates (Millipore, Schwalbach, Germany) were coated overnight with the capture antibodies in sterile PBS. All antibodies were obtained from BD Pharmingen, San Diego, CA. The following coating antibodies were used: R4-6A2 was used for IFN-γ, JES6-1A12 was used for IL-2 and TC11-18H10 was used for IL-17. Plates were blocked for 1 h with sterile PBS/BSA 0,5% and washed 3× with sterile PBS. Splenocytes were plated in HL-1 medium in triplicate cultures each. Spinal cord single cell suspensions were pooled from 3 to 4 animals and plated in HL-1 medium in duplicate wells. Thereafter cells were stimulated with PLPp (final concentration of 20 μg/ml), with CpG (final concentration of 10 μg/ml) or with both agents. Subsequently plates were incubated at 37°C, 5% CO_2 _for 16 h. After washing with PBS followed by PBS/BSA 0,5%, detection antibodies were added overnight. Biotinylated XMG1.2 was used for IFN-γ, biotinylated JES6-5H4 was used for IL-2, biotinylated TC11-8H4.1 was used for IL-17. The plate bound second antibody was then visualized by adding streptavidin-alkaline phosphatase (SAV-AP, DAKO, Glostrup, Denmark) and NBT/BCIP substrate (Bio-Rad, Munich, Germany). Image analysis of ELISPOT assays was performed with the ImmunoSpot™ Analysis Software after scanning the plates with an ImmunoSpot™ Analyzer (CTL-Europe, Bonn, Germany). In brief, digitized images of individual wells of the ELISPOT plates were analyzed for cytokine spots, based on the comparison of experimental wells (containing immune cells and stimuli) and control wells (immune cells, no stimuli). After separating spots that touched or partially overlapped, non-specific 'background noise' was gated out by applying spot size and circularity analysis as additional criteria. Then, spots that fell within the accepted criteria were highlighted and counted. Single wells which could not be enumerated because of confluence phenomena were assessed by using the highest numbers of cytokine-producing cells which could be regularly counted in other wells in the same assay as an approximated estimate.

### Statistical analysis

For statistical analysis, the two-sided *t*-test for different variances (GraphPad PRISM 4 software, La Jolla, USA) was used. Differences at *p *< 0.05 were considered statistically significant.

## Results

### Injection of SJL mice with PLPp/CPG/IFA does not result in clinical disease

**Figure 1:**
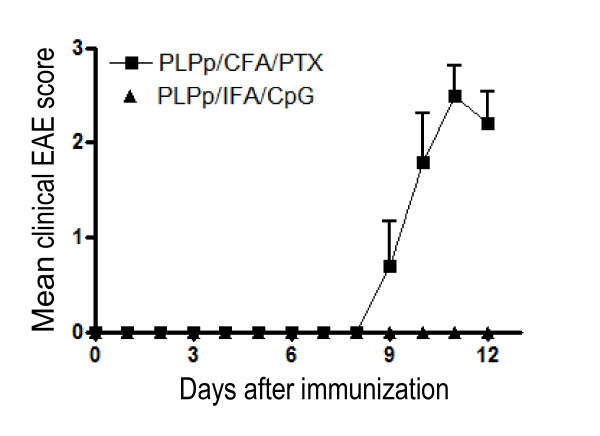
**Representative disease course of SJL mice**. 5 mice were immunized with PLPp/CFA with addition of pertussis toxin. 5 mice were immunized with PLPp and CpG in IFA. The mean clinical EAE score is shown with standard deviation. The differences in disease scores between the two groups on days 10, 11 and 12 were highly significant (p < 0.001).

To study the impact of TLR9 stimulation during the priming of the autoreactive T cell response, we immunized SJL mice with PLPp emulsified in CpG/IFA (PLPp/CpG/IFA). In contrast to the mice immunized according to the standard protocol at the same time, no clinical signs of EAE were observed (Figure 1), consistent with previously reported results by others [21;22]. No signs of EAE were seen during the entire observation period of 90 days. SJL mice immunized with PLPp in CFA and 2 subsequent applications of PTX (PLPp/CFA/PTX) developed severe EAE.

### Injection of SJL mice with PLPp/CPG/IFA results in a PLPp-specific Th1 response, but not a Th17 response

**Figure 2:**
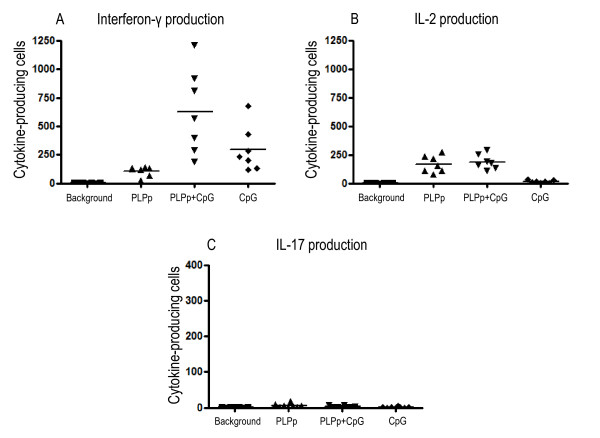
**Frequencies of IFN-γ (A), IL-2 (B) and IL-17 (C) producing cells in spleen cells of animals immunized with PLPp in CpG/IFA after stimulation with PLPp, CpG or combined activation**. Spleens were isolated on day 14 after immunization, and single cell suspensions were made as described in *Materials and Methods*. Cells were plated at a density of 1.000.000 cells per well. The cumulative data of 7 animals tested individually in two independent experiments are shown. Measurements were conducted in triplicate wells each. Each data point indicates one individual animal, lines represent the mean. *p *values are given in Section 3.

To investigate whether autoantigen specific T lymphocytes are present in the peripheral lymphatic tissue of mice immunized with PLPp_/_CpG/IFA, measurements of cytokine expression (IFN-γ, IL-2, IL-17) in the spleens of animals were conducted by ELISPOT at day 14 after immunisation. High numbers of IFN-γ- (mean: 104.67, SD: 42.82) and IL-2- (mean: 168.72, SD: 73.44) producing cells were identified after restimulation with PLPp (Figure 2A and 2B). Combined stimulation with PLPp and CpG led to strong up-regulation of IFN-γ production (mean: 626.31, SD: 370.19) in comparison to stimulation with PLPp alone (p < 0.01), but the number of IL-2 producing cells (mean: 188.28, SD: 64.79) did not change significantly. Almost no IL-17 producing cells (mean: 4.19, SD: 3.22) were present in the spleens of mice immunized according to the alternative protocol (Figure 2C). Additional stimulation with CpG did not lead to induction of higher numbers of IL-17 secreting cells (mean: 2.33, SD: 2.52).

### CpG enhance PLPp-specific IFN-γ production in spleens of mice during acute EAE, but have no effect on PLPp-specific IL-17 production

**Figure 3:**
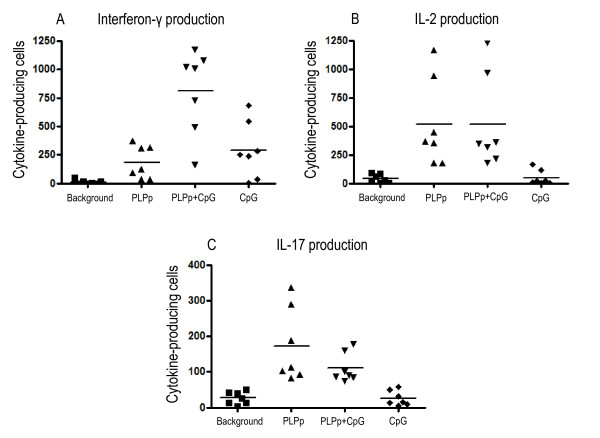
**Frequencies of IFN-γ (A), IL-2 (B) and IL-17 (C) producing cells in spleens of animals immunized with PLPp in CFA and pertussis toxin after stimulation with PLPp, CpG or combined activation**. Spleens were isolated on day 12 after immunization, and single cell suspensions were made as described in *Materials and Methods*. Cells were plated at a density of 1.000.000 cells per well. The cumulative data of 7 animals tested individually in two independent experiments are shown. Measurements were conducted in triplicate wells each. Each data point indicates one individual animal, lines represent the mean. *p *values are given in Section 3.

To assess the impact of TLR 9 ligation on PLPp-specific cytokine responses in the spleens of mice immunized according to the standard (PLPp/CFA/PTX) protocol, we conducted measurements on splenocytes of mice sacrificed on day 12 after EAE induction. High frequencies of IFN-γ- (mean: 183.24, SD: 143.62) and IL-2-producing (mean: 519.09, SD: 384.08), PLPp-specific T cells could be observed in spleens of mice during acute EAE after recall with PLPp. The presence of CpG had a considerable superadditive effect on the number of IFN-γ-producing cells (mean: 809.43, SD: 367.45), p = 0.0012 for PLPp vs. PLPp+CpG), but no effect on IL-2 production (mean: 519.48, SD: 415.49) (Figure 3). These findings are comparable to cytokine responses of splenocytes isolated out of mice immunized with PLPp/CpG/IFA at day 14. ELISPOT measurements showed that splenocytes of mice, in which EAE was successfully induced, produced IL-17 after restimulation with PLPp (mean: 172.33, SD: 103.68). However, CpG did not increase the number of IL-17-producing, antigen specific T cells (mean: 110.67, SD: 40.99) in splenocytes.

### CpG potentiate both autoantigen-specific IFN-γ and IL-17 production locally in the CNS during acute EAE

**Figure 4:**
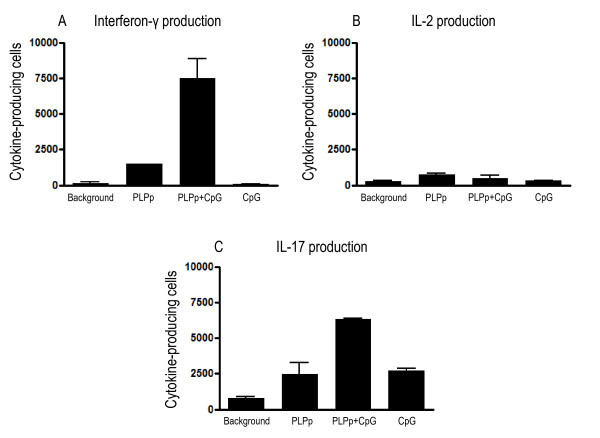
**Frequencies of IFN-γ (A), IL-2 (B) and IL-17 (C) producing PLPp specific cells in spinal cords of animals immunized with PLPp in CFA and pertussis toxin after stimulation with PLPp, CpG or combined activation**. Spinal cords were isolated on day 12 after immunization, and single cell suspensions were made as described in *Materials and Methods*. Cells were plated at a density of 100.000 cells per well. Raw data is adjusted to 1.000.000 cells per well for a direct comparison with frequencies of cytokine-producing spleen cells. Each bar represents two independent experiments with pooled spinal cord cultures of totally 7 mice (4 mice in the first experiment, 3 mice in the second experiment) due to the small number of mononuclear cells which can be recovered the CNS. Measurements were conducted in duplicate wells each. *p *values are given in Section 3.

To study the impact of microbial components on cytokine responses in the CNS in acute EAE, pooled spinal cord single cell suspensions from mice with acute EAE at day 12 were restimulated with PLPp and CpG and the frequencies of cytokine-producing cells were determined by ELISPOT (Figure 4). Without antigen stimulation, residual IL-17 (mean: 780, SD: 197.99) and IFN-γ (mean: 145, SD: 148.49) production was detected, reflecting the activity of primed T cells *in situ *stimulated by endogenous antigen. With external PLPp stimulation, dominant IL-17 production (mean: 2465, SD: 1124.3) was detected, accompanied by IFN-γ (mean: 1505, SD: 7.07) and (less) IL-2 (mean: 755, SD: 77.78) production. Additional stimulation with CpG led to a strong upregulation of IFN-γ secretion in the target organ (mean: 7485, SD: 1378.86, p < 0.01 for PLPp vs. PLPp+CpG), as well as a moderate upregulation of IL-17 production (mean: 6320, SD: 56.57, p = 0.01 for PLPp vs. PLPp+CpG), but had no effect on IL-2 production (mean: 515, SD: 219.2, p = ns). Stimulation with CpG only (without addition of external PLPp) led to strong selective enhancement of IL-17 production (mean: 2715, SD: 261.63, p = 0.014 for CpG vs. background), but did not affect spontaneous IFN-γ (mean: 85, SD: 35.36, p = ns) or IL-2 (mean: 315, SD: 49.5, p = ns) production of the inflammatory CNS isolate. As a consequence, the IFN-γ/IL-17 ratio substantially differed depending on the type of stimulation (background: 0.19, PLPp: 0.61, PLPp+CpG: 1.18, CpG: 0.03).

### CpG oligonucleotides do not essentially alter the encephalitogenicity of in-vitro-stimulated PLPp-specific T cells pre-primed in the immune periphery

**Figure 5:**
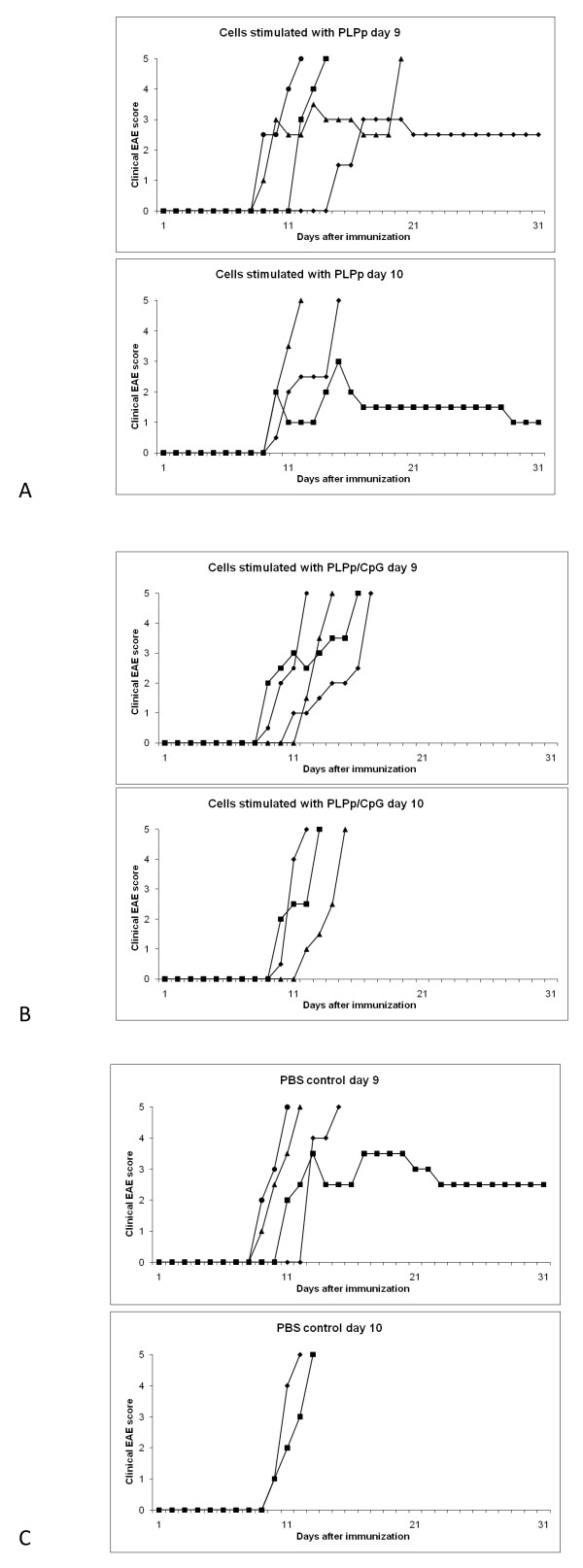
**Adoptive transfer of PLPp-stimulated and PLPp/CpG-stimulated PLPp-specific T cells into SJL mice with actively induced EAE**. Female SJL mice injected with PLPp/CFA and PTX as described above were (either at day 9 or day 10) injected with cells re-stimulated *in vitro *either with PLPp (A) or PLPp/CpG (B). A control group received PBS at day 9 or day 10, respectively (C). The disease course of all animals is depicted individually. Further details are described in the results section.

To investigate the impact of additional CpG stimulation on pre-primed PLPp-specific T cells *in vitro*, adoptive transfer experiments were conducted. Female SJL mice were immunized with PLPp/CFA and PTX with the standard protocol described above. On day 7 after immunization (when the immune response in the periphery was well established) these mice were sacrificed and their spleen cells were taken in culture (either with PLPp or with PLPp/CpG). After three days of culture, the remaining viable cells were transferred into recipient female SJL mice which also had been immunized with PLPp/CFA and PTX with the standard protocol. To get more differentiated information, one set of animals was injected at day 9 after immunization (when disease just started in some animals), another set was injected at day 10 after immunization (when disease was already established). A control group at day 9 and day 10 each received PBS only, no cells. As can be seen from Figure 5A-C, disease was quite severe in all groups in this set of experiments, and even in the control group most animals eventually died after a fairly short time (the disease course of all animals is depicted individually). In both groups which received cells restimulated with PLPp one animal each survived (in contrast to the respective group receiving cells restimulated with PLPp/CpG). This could (carefully) be interpreted as further evidence for the potential of CpG to enhance the encephalitogenicity of already primed PLPp-specific T cells. However, there was not a fundamental alteration of disease into a negative or positive fashion affecting the majority of animals.

## Discussion

The principal goal of this study was to investigate how a microbial immune stimulant associated with a systemic infection can influence the cytokine signature of an ongoing autoimmune reaction in CNS autoimmune disease. The focus hereby was (1) on potential differences between immune periphery and the CNS and (2) on distinct effects regarding Th1 and Th17 cells. The human clinical situation to which the results of our EAE-based study might apply is the mechanism via which a systemic infection might worsen ongoing CNS autoimmune pathology in MS and thereby cause disease progression or relapse [6-9]. As a paradigmatic microbial stimulus simulating "infection" in our study CpG oligonucleotides were investigated regarding their co-stimulating effect on the PLPp-specific cytokine signature in PLPp-induced EAE in SJL mice. We concentrated on the situation in acute EAE by measuring the quantity of PLPp-specific IFN-γ-, IL-2- and IL-17-producing cells in the immune periphery and the target organ CNS. The presence of microbial factors, in particular TLR stimulants, can have different effects on the immune system when it happens initially during the priming of the autoimmune reaction [22-24] in contrast to its appearance during the actual autoimmune disease [11;12;25;26]. It has been demonstrated by two previous studies that mice injected with PLPp/CPG do not develop clinical EAE, but a strong PLPp-specific Th1 response [21;22]. Our data confirms these observations, as well as the fact that there is a complete lack of a PLPp-specific Th17 response after this injection protocol. In contrast to one of these previous works [22] however, we want to avoid the simplistic conclusion that the mere absence of Th17 cells explains the lack of clinical disease symptoms. The same group which has initially described the adjuvant effect of CpG in EAE [21] has demonstrated that EAE can be induced in the absence of Th17 cells, and that Th1 cells cannot automatically be seen as protective, but induce a different type of CNS pathology than do Th17 cells [15].

Under the conditions of established CNS-autoantigen-specific immunity, CpG oligonucleotides effectively enhance PLPp-specific IFN-γ production in splenocytes, i.e. the immune periphery. Since they also trigger IFN-γ without PLPp, this appears to be (partially) a stimulating effect on the non-T-cell compartment, presumably monocytes and macrophages. However, combined stimulation (PLPp and CpG) results in an over-additive effect, which is more than the sum of the single stimulations with PLPp or CpG alone. This is not dependent on the presence of clinical EAE symptoms - the effect is the same both in healthy animals after injection with PLPp/CpG/IFA and in animals with severe EAE after injection with PLPp/CFA and PTX. No effect is seen in both animal groups on PLPp-specific IL-2 and IL-17 production, neither alone or with combined stimulation. Therefore, the effect of TLR9 ligation on the autoreactive T cell population in the periphery does not clarify its disease-enhancing effect in EAE which has been described by others [11]. In contrast, in the inflamed CNS a complex picture emerges: Already stimulation with CpG alone (without addition of PLPp) triggers strong IL-17 production, but no IFN-γ production. With combined stimulation (CPG and PLPp), a strong enhancement of both IFN-γ and IL-17 occurs, whereas IL-2 production is not essentially altered at any stimulation condition. Since in the spinal cord, which was isolated directly *ex vivo*, considerable amounts of endogenous antigen are likely to be presented by local APC, there is most likely no pure TLR9 stimulation, but also combined stimulation when only CpG are experimentally applied. This explains why there is already considerable residual cytokine production without external stimulant, with IL-17 being the dominant cytokine. CpG might enhance the already existing Th17 response, but might also activate cells of the innate immune system which are able to produce IL-17. In any case, these cells are in EAE only present in the inflamed CNS, not in the immune periphery, or they can only be activated in the inflamed CNS. This indicates that one immunological stimulus (CpG) can have substantially different effects on the cytokine architecture in distinct stages of an autoimmune reaction. TLR9 stimulation alone during priming is not sufficient to induce antigen-specific Th17 cells, but has a decisive effect on IL-17 production in the inflamed target organ *in situ *under various local conditions. This is paralleled by the observation that *in vitro *co-stimulation of peripherally primed PLPp-specific T cells with CpG does not essentially alter the encephalitogenic potential of these cells in adoptive transfer experiments.

As a main conclusion of this set of data, within the CNS, microbial stimulants might cause very heterogeneous and patchy micro-effects. Both the presence of an infectious stimulus and the amount of CNS autoantigen locally present might determine the IFN-γ/IL-17 ratio which results. External addition of PLPp (which might yield unrealistic concentrations which do not occur *in vivo*) causes a strongly different IFN-γ/IL-17 ratio than the culture with endogenous PLPp only. A landmark study has suggested that the local IFN-γ/IL-17 ratio in the individual lesions determines disease progression and depends on the anatomic site of the lesion [27]. Therefore, different inflammatory lesions in the same CNS might be differentially affected by the presence of a microbial stimulus, depending on autoantigen availability and other local factors. This might be in line with the heterogeneity of MS lesions in one and the same patient. For the potential connection between infection and worsening of already ongoing CNS autoimmune disease, our results suggest that the crucial effect of inflammatory mediators is achieved in the CNS *in situ*. A scenario is possible where traces of low molecular pathogenic factors present in abundance in peripheral compartments (e.g. blood, spleen, lymphatic tissues) during a systemic inflammation enter the brain and lead in cooperation with local ongoing autoimmune processes to reactivation of quiescent or suppressed autoantigen-specific T cells. These activated memory cells recruit other antigen-specific and bystander immune cells from the peripheral pool causing thereby an acute relapse or disease progression. This concept is supported by observations by other groups [11;12;25]. One of these studies even claims that endogenous, non-microbial TLR9 ligands like phosphodiester DNA (which might arise in the course of the autoimmune process) might substitute for microbial TLR9 ligands and thereby further local autoimmune pathology [11].

Of note, it is not the purpose of our study to support argumentation for a unidimensional pathogenic role for one single cytokine, neither for IL-17 nor for IFN-γ. In the light of the current literature on EAE and other experimental autoimmune diseases, different cytokines can induce different types of autoimmune pathology [15;16;28]. We conclude that (1) CpG can have different effects on T cell cytokine production depending on whether they are present in the priming phase or during the ongoing autoimmune response (2) the effects in the immune periphery and in the inflamed CNS differ qualitatively and quantitatively and (3) in the CNS, conditions of the local microenvironment like the amount of antigen additionally determine the cytokine signature. An organism with an ongoing autoimmune response in which additionally infectious agents and their microbial stimuli are present is likely to be an anatomically quite heterogeneous "mosaique" regarding the cytokines which dominate ongoing local autoimmunity, in particular in the inflamed target organ. Simple mechanistic extrapolations towards the human disease MS currently therefore do not seem justified, in particular not for one single molecular receptor or ligand. Additional studies have to clarify which cellular compartments in the CNS are producing the respective cytokines and how they change under the influence of exogenous/endogenous TLR ligands during the various stages of the course of the autoimmune disease.

## Acknowledgements

HHH and VS were supported by a Grant of the Forschungskomission der Medizinischen Fakultät der Universität Düsseldorf (Nr. 9772322). HHH was also supported by grants of the Deutsche Forschungsgemeinschaft (Ho 4392/1-1), of the Strategischer Forschungsfonds der Universität Düsseldorf and of the Deutsche Multiple Sklerose Gesellschaft. BH and PV were supported by a Grant of the Deutsche Forschungsgemeinschaft (He 2386/7-1). The MS center at the Department of Neurology, Heinrich-Heine-University is supported in part by the Walter-and-Ilse Rose Stiftung.

## Competing interests statement

The authors declare that they have no competing interests.

## Authors' contributions

HHH designed the study, analyzed data, wrote the paper and performed experiments, VS analyzed data, wrote the paper and performed experiments, TD, AKM and PV wrote the paper and performed experiments, BCK, BH and HPH wrote the paper. All authors read and approved the final manuscript.
